# Pertrochanteric fracture of the femur in the Finnish National Hospital Discharge Register: validity of procedural coding, external cause for injury and diagnosis

**DOI:** 10.1186/1471-2474-15-98

**Published:** 2014-03-24

**Authors:** Tuomas T Huttunen, Pekka Kannus, Harri Pihlajamäki, Ville M Mattila

**Affiliations:** 1 Department of Anesthesia, Tampere University Hospital, Tampere, Finland; 2 Department of Orthopedics and Trauma Surgery, Tampere University Hospital, Tampere, Finland; 3 UKK Institute for Health Promotion Research, Injury & Osteoporosis Research Center, Tampere, Finland; 4 Medical School, University of Tampere, Tampere, Finland; 5 Division of Orthopedics and Trauma Surgery, Seinäjoki Central Hospital, Seinäjoki, Finland; 6 University of Tampere, Seinäjoki, Finland; 7 Department of Clinical Science, Intervention and Technology, Karolinska Institutet, Stockholm, Sweden; 8 Division of Orthopedics and Biotechnology, Karolinska Institutet, Stockholm, Sweden; 9 Department of Orthopedics, Karolinska University Hospital, Stockholm, Sweden; 10 Simppoonkatu 5 B 8, FIN-33230 Tampere, Finland

## Abstract

**Background:**

Hospital discharge data is routinely collected in Finland and it is an invaluable source of information when assessing injury epidemiology as well as treatment. The database can be used when planning injury prevention and redirecting resources of the health care system. Most recently our hospital discharge register has been used to assess the incidence of surgical treatment of common fractures. This study was aimed to evaluate the coverage and accuracy of the Finnish National Hospital Discharge Register (NHDR) focusing on hip fractures. In other words, patients hospitalized for a pertrochanteric hip fracture were used to assess the validity of the NHDR.

**Methods:**

The validity of the NHDR was assessed by comparing the data in hospital discharge register with the original patient records and radiographs in three separate hospitals; Tampere University Hospital, Hatanpää City Hospital of Tampere, and the Central Hospital of Kanta-Häme. The study analysis included 741 patients hospitalized due to pertrochanteric hip fracture between 1^st^ January 2008 and 31^st^ December 2010.

**Results:**

The diagnosis was correctly placed on 96% (95% CI: 94 to 97%) of the 741 patients when radiographs were used as golden standard. The procedural coding had coverage of 98% (95% CI: 96 to 98%) and an accuracy of 88% (95% CI: 85 to 90%). The coverage of the external cause for injury was found to be 95% (95% CI: 94 to 97%) with an accuracy of 90% (95% CI: 87 to 92%).

**Conclusions:**

Our results show that the validity of the Finnish NHDR is excellent as determined by accuracy of diagnosis and both accuracy and coverage of procedural coding and external cause for injury. The database can be used to assess injury epidemiology and changes in surgical treatment protocols.

## Background

The National Hospital Discharge Register (NHDR) is statutory, computer-based register in Finland including all hospitalization data in the country since 1967. Currently it contains data on almost 70 variables including a personal identification number, age, sex and domicile of the subject, place and cause of injury, duration of hospital stay, diagnoses, and procedures performed during the hospital stay.

The Finnish NHDR has been valuable and widely used in epidemiological studies on sports and other injuries, and more recently when describing incidence of surgical treatment [1-5]. A few studies have investigated the validity, i.e. coverage and accuracy of the register with regard to diagnosis [6-9]. In a recent review, Sund compiled all studies concerning the quality of the Finnish NHDR and found it to be a valid source of information [10].

We previously investigated the coverage and accuracy of diagnosis in the Finnish NHDR and found that concerning the diagnosis of the cruciate ligament injury of the knee the NHDR had coverage of 92% and an accuracy of 89% [8]. Other previous studies observed that the accuracy of pelvic and hip fracture diagnoses ranged from 95% to 98% [6,7].

As noted above, in addition to the diagnoses placed during the hospitalization, the Finnish NHDR contains information on procedural coding, external cause for injury and type of injury or accident. However, when conducting register-based epidemiological studies on injuries it is of utmost importance that coverage and accuracy of these variables are well assessed.

Currently, studies on the validity of procedural coding and external cause for injury are scarce [6], and to our knowledge no previous study has focused on assessing the validity of procedural coding after implementation of the Nomesco (Nordic Medico-Statistical Committee) procedure classification in 1996.

In this study we assessed the validity of the Finnish NHDR in relation to the diagnosis, procedural coding and external cause for one specific injury (pertrochanteric hip fracture) by determining the coverage and accuracy of these parameters in three Finnish hospitals. Pertrochanteric hip fracture was considered suitable for our purposes because it is a common, surgically treated injury.

## Methods

The study sample included three level one to three trauma hospitals in Finland: Tampere University Hospital (level I), Hatanpää City Hospital of Tampere (level III), and the Central Hospital of Kanta-Häme (level II). Patients 18 years or older were included. In Finland, register-based studies do not require the approval of ethics committee by legislation. However, all register studies that utilize any confidential medical information such as patient charts and radiographs, require an approval of the corresponding institution or hospital. These permissions were obtained from all hospitals that participated our study.

The sample was obtained by selecting from the NHDR all patients with a diagnosis of pertrochanteric hip fracture admitted alive to any of the three study hospitals between 1^st^ of January 2008 and 31^st^ of December 2010. All re-hospitalizations due to either rehabilitation, medical or surgical complications were excluded based on the original medical records and thus only primary hospitalizations after the initial injury were included into the study. We used the International Classification of Diseases 10^th^ edition (ICD-10) code S72.1 [11]. After 1996 all procedural coding in Finland has been done according to a Finnish version of Nomesco procedure classification and so Nomesco procedural coding was used in this study. The main outcome variable in the study was assessed by comparison of data from the NHDR to the original patient charts and x-ray archives.

As noted above, the Finnish NHDR is a mandatory national register for all of our hospitals encompassing private, public, and other institutions. The NHDR is collected and maintained by the National Institute for Health and Welfare, Helsinki, Finland.

After the sample was collected, all selected cases were evaluated by going through the patient chart, and x-rays taken both pre- and post-operatively. Accuracy of the diagnosis was assessed by examining pre-operative x-rays and determining the type of the hip fracture (fracture of the femoral neck, pertrochanteric fracture or subtrochanteric fracture) and then comparing the result to the type of the fracture (diagnosis) recorded in the hospital register. Coverage of the procedure coding was determined by reading through the medical records and radiographs to find the patients who had undergone surgery. It was then determined how many of these procedures were recorded into the NHDR. Accuracy (dichotomy right/wrong) of the procedural coding was assessed by examining the post-operative x-rays and determining the type of fixation used and then comparing to the type of fixation (procedure code) in the NHDR.

Coverage of the external cause for injury was examined by comparing the number of patients who were injured (and had a diagnosis of a pertrochanteric fracture) to the number of patients who had an external cause for injury recorded on the hospital discharge register. Accuracy of the external cause for injury was assessed by going through the medical records and determining the mechanism of injury (for example a fall) and then comparing it to the external cause for injury recorded on the hospital register.

All of the results were expressed as a percentage with 95% confidence interval (CI).

Two experienced physicians (T.T.H. and V.M.M.) examined the patient charts and radiological findings separately. In case of a disagreement, consensus was reached. If there was an unresolved radiological finding, the result was resolved by the expert opinion of the radiologist who had originally evaluated the radiological images.

## Results

According to the sample taken from the above noted three individual hospitals a total of 1,112 hospitalizations with a primary or secondary diagnosis of pertrochanteric hip fracture were identified during the study period. As only the primary hospitalizations were included into the study sample comprised 741 cases. Majority of patients were female (n = 509, 69%) The mean age of patients was 81 years. Men were younger (mean age 76 years) than their female counterparts (mean age 83 years).

Most (n = 729, 98%) of the 741 patients with a pertrochanteric hip fracture were operated on. Of the non-surgically treated patients (n = 12, 2%), two refused surgical treatment and 10 died prior surgery.

A pertrochanteric hip fracture was coded as diagnosis in all of the 741 (100%) patients (inclusion criterion). According to the radiological assessment, the NHDR diagnosis was accurate on 709 of the 741 patients resulting in an accuracy of the diagnosis of 96% (95% CI: 94 to 97%). The remaining 32 fractures were falsely registered: 24 (75%) of these were actually fractures of the neck of the femur, 5 (16%) were subtrochanteric hip fractures and 3 (9%) were fractures of the shaft of femur.

A procedural code was found on 711 of the 729 patients of who had undergone a surgical procedure. The coverage of the procedural coding was therefore 98% (95% CI: 96 to 98%). The reasons for not registering a surgical procedure into the NHDR were not uniform and therefore we were unable to further categorize these 18 cases. Most commonly the reason was that the patient was transferred from the surgical ward into another ward because of a medical reason (for instance after suffering heart failure or infection) and the surgical procedure was later performed while being on a non-surgical ward.

Of the 711 patients with a procedural code recorded on the NHDR, 10 died soon after the surgery and did not have x-rays taken after the operation. Therefore it was not possible to validate these cases. Of the remaining 701 patients 616 had a correct code. The accuracy of the procedural coding was therefore 88% (95% CI: 85 to 90%). The remaining 85 procedures were erroneously registered into the NHDR. Internal fixation of fracture of neck of femur with nail or screw (NFJ50) was wrongly used in 1 case. Internal fixation of fracture of upper part of femur with sliding hip screw (NFJ52) was wrongly used in 24 cases. Internal fixation of fracture of upper part of femur with trochanteric nail (NFJ54) was wrongly used in 57 cases. Internal fixation of fracture of other parts of femur (NFJ62) was wrongly used in 3 cases. The most common errors were to mix procedures NFJ52 and NFJ54.

An external cause for injury was registered on 707 of the 741 patients resulting in coverage of 95% (95% CI: 94 to 97%). Of these 707 patients with an external reason for injury 635 had a correct code registered on the NHDR resulting in an accuracy of 90% (95% CI: 87 to 92%).

## Discussion

Data collection in Finnish hospitals is statutory and therefore performed routinely for all hospital admissions. The collected data should be readily usable for epidemiological research and in fact numerous reports have been published utilizing this data. However, no previous research has been made in attempt to validate the coverage and accuracy of the procedural coding in the NHDR, although in general the coverage and accuracy of diagnosis in the Finnish NHDR have been found to be good [10].

In this study we assessed the validity of diagnosis, procedural coding and external cause for injury in hospitalizations after a pertrochanteric fracture of the femur. Accuracy of diagnosis and both coverage and accuracy of procedural coding and external cause for injury were found to be excellent.

Pertrochanteric hip fracture was selected because practically all cases undergo surgery and therefore result in hospitalizations with procedure coding registered into the NHDR. Furthermore, according to the recommendations of use of the ICD-10, whenever injury coding (S00-T98) is used it is obligatory to use the external causes for morbidity and mortality (V01-Y98) to try and classify the environmental events and circumstances leading to the injury [11]. Thus, in our study during one hospitalization period it was possible to assess information of the use of these three individual variables.

Pertrochanteric hip fracture is an injury that requires swift treatment [12]. It is common practice in Finland that surgeons dictate the course of procedures including procedural coding after the operation. It is possible that work done during uncomfortable hours increases the inaccuracy of procedural coding. There are no studies comparing the validity of procedural coding in the NHDR between elective and acute surgical procedures. Pertrochanteric hip fracture can be surgically treated by using various surgical approaches, which also increases the risk for inaccuracy. In this regard our results of 88% accuracy of procedural coding can be considered excellent.

In our previous study on validity of anterior cruciate ligament injury diagnosis, we found the diagnosis coding to have both good coverage (92%) and accuracy (89%) [8]. In addition, Luthje and coworkers took a sample from the NHDR in 1988 by selecting all patients (n = 1,212) hospitalized due to pelvic fracture [7]. They then validated the diagnosis by selecting every tenth case randomly and reviewing the case medical records against the data on the NHDR. Accuracy of diagnosis was found to be very high, 97%.

Our current result on accuracy of diagnosis of 96% is well in line with our own previous results with anterior cruciate ligament injury (92%) and with the findings of Luthje and coworkers (97%). The true coverage of the diagnosis could not be assessed in our present study, as the diagnosis code S72.1 was a selection criterion for the study population. Assessment of coverage of diagnosis would have required a different study setting and therefore our principal aim was to assess the coverage and accuracy of procedural coding.

We are not aware of any recent study trying to assess accuracy of diagnosis and both accuracy and coverage of procedural coding and external cause for injury in the Finnish or any other NHDR. In a Norwegian study, Loftus and coworkers found that there were major variations in registering diagnosis in different hospital discharge registers and questioned their reliability [13]. In a recent review, Ludvigsson et al. found that the Swedish National Inpatient Register had excellent injury diagnosis coding with a high reporting rate for external cause for injury [14]. In Finland Keskimäki and Aro took a random sample of 2,285 cases from the Finnish NHDR including all different reasons for hospitalizations in the year 1986 and compared this data to the individual medical records. The diagnosis was found to be accurate in 95% of cases but procedural coding was quite inaccurate (70-78%). Coding for external cause for injury was also poorly placed (64%). It has to be remembered, however, that the study was done in 1986 and the register used the ICD-8. The development of the NHDR, introduction of the ICD-10 coding system and better schooling in their use are likely to explain the major differences to our current results.

Although orthopedic surgeons may concentrate more on outcomes of surgical procedures, the validity of the NHDR could be increased further by personal feedback and education in using the NHDR. Thus, we suggest that more resources should be put into education and proper use of procedural coding including surgeons in training. This will help in assessing the future trends in common musculoskeletal injuries and their treatment.

## Conclusions

We found that the accuracy of diagnosis and both accuracy and coverage of procedural coding and external cause for injury in the Finnish National Hospital Discharge Register are excellent. The register can be used as a reliable source of information especially in epidemiological studies of injuries.

## Competing interests

The authors declare that they have no competing interests.

## Authors’ contributions

TH and VM contributed in all stages of the study. PK and HP contributed in study design, data interpretation and writing the final manuscript. All authors read and approved the final manuscript.

## Pre-publication history

The pre-publication history for this paper can be accessed here:

http://www.biomedcentral.com/1471-2474/15/98/prepub

## References

[B1] Huttunen TT Kannus P Lepola V Pihlajamaki H Mattila VM Surgical treatment of humeral-shaft fractures: a register-based study in Finland between 1987 and 2009 Injury 2012 43 10 1704 1708 10.1016/j.injury.2012.06.011 22771121

[B2] Huttunen TT Launonen AP Pihlajamaki H Kannus P Mattila VM Trends in the surgical treatment of proximal humeral fractures - a nationwide 23-year study in Finland BMC Musculoskelet Disord 2012 13 261 23273247 10.1186/1471-2474-13-261 PMC3537526

[B3] Korhonen N Niemi S Parkkari J Sievanen H Kannus P Incidence of fall-related traumatic brain injuries among older Finnish adults between 1970 and 2011 JAMA 2013 309 18 1891 1892 10.1001/jama.2013.3356 23652518

[B4] Mattila VM Huttunen TT Sillanpaa P Niemi S Pihlajamaki H Kannus P Significant change in the surgical treatment of distal radius fractures: a nationwide study between 1998 and 2008 in Finland J Trauma 2011 71 4 939 942 discussion 942–933 10.1097/TA.0b013e3182231af9 21986738

[B5] Mattila VM Huttunen TT Haapasalo H Sillanpaa P Malmivaara A Pihlajamaki H Declining incidence of surgery for Achilles tendon rupture follows publication of major RCTs: evidence-influenced change evident using the Finnish registry study Br J Sports Medin press 10.1136/bjsports-2013-092756 24128757

[B6] Keskimäki I Aro S Accuracy of data on diagnosis, procedures and accidents in the Finnish Hospital Discharge Register Int J Health Sci 1991 2 15 21

[B7] Luthje P Nurmi I Kataja M Heliovaara M Santavirta S Incidence of pelvic fractures in Finland in 1988 Acta Orthop Scand 1995 66 3 245 248 10.3109/17453679508995533 7604706

[B8] Mattila VM Sillanpaa P Iivonen T Parkkari J Kannus P Pihlajamaki H Coverage and accuracy of diagnosis of cruciate ligament injury in the Finnish National Hospital Discharge Register Injury 2008 39 12 1373 1376 10.1016/j.injury.2008.05.007 18703187

[B9] Leppala JM Virtamo J Heinonen OP Validation of stroke diagnosis in the National Hospital Discharge Register and the Register of Causes of Death in Finland Eur J Epidemiol 1999 15 2 155 160 10.1023/A:1007504310431 10204645

[B10] Sund R Quality of the Finnish Hospital Discharge Register: a systematic review Scand J Public Health 2012 40 6 505 515 10.1177/1403494812456637 22899561

[B11] World Health Organization International Statistical Classification of Diseases and Related Health Problems 2010 10 Geneva: World Health Organization

[B12] Orosz GM Magaziner J Hannan EL Morrison RS Koval K Gilbert M McLaughlin M Halm EA Wang JJ Litke A Silberzweig SB Siu AL Association of timing of surgery for hip fracture and patient outcomes JAMA 2004 291 14 1738 1743 10.1001/jama.291.14.1738 15082701 PMC1454713

[B13] Lofthus CM Cappelen I Osnes EK Falch JA Kristiansen IS Medhus AW Nordsletten L Meyer HE Local and national electronic databases in Norway demonstrate a varying degree of validity J Clin Epidemiol 2005 58 3 280 285 10.1016/j.jclinepi.2004.07.003 15718117

[B14] Ludvigsson JF Andersson E Ekbom A Feychting M Kim JL Reuterwall C Heurgren M Olausson PO External review and validation of the Swedish National Inpatient Register BMC Public Health 2011 11 450 10.1186/1471-2458-11-450 21658213 PMC3142234

